# Effect of Diaphragm Plication in Thymoma Patients with and without Myasthenia Gravis

**Published:** 2018-03

**Authors:** Reza Bagheri, Seyed Ziaollah Haghi, Reza Afghani, Vahab Azmounfar, Saeed Hakimian, Mohammad Baradaran Firoozabadi, Negar Morovatdar, Elham Lotfian

**Affiliations:** 1 Lung Disease Research Center, Mashhad University of Medical Sciences, Mashhad, Iran; 2 Department of General Surgery, 5th of Azar Hospital, Faculty of Medicine, Golestan University of Medical Sciences, Gorgan, Iran; 3 Student Research Committee, Mashhad University of Medical science, Mashhad, Iran; 4 Mashhad University of Medical Science, Mashhad, Iran; 5 Imam Reza Clinical Research Unit, School of Medicine, Mashhad University of Medical Sciences, Mashhad, Iran

**Keywords:** Plication, Diaphragm, Phrenic nerve, Thymoma, Myasthenia gravis

## Abstract

**Background:**

Thymoma is the most common tumor of the anterior mediastinum that has the most effective treatment, as it can be completely resected. In patients with advanced stage, phrenic nerve involvement can be seen and suggested treatment for these patients is unilateral phrenic excision and diaphragm plication. However in patients with myasthenia gravis, there are concerns in relation to this method of treatment. The aim of this study is to evaluate the effects of plication of the diaphragm on complications of phrenic nerve excision in thymoma patients with and without myasthenia gravis involving the phrenic nerve.

**Materials and Methods:**

A retrospective cohort study was performed on 26 patients with thymoma; half of the patients had myasthenia gravis and the other half did not have myasthenia gravis. We performed diaphragm plication in 7 patients in each group with excision of phrenic nerve. Patients were evaluated based on preoperative and postoperative variables.

**Results:**

The patients' age (P=0.943), sex (P=0.999), blood loss during surgery (P=0.919), need for transfusion during surgery (P=0.999), short term complications (P=0.186), need for tracheostomy (P=0.27) and mortality (P=0.09) differences were not significant. However, the average duration of ICU stay (P=0.001) and intubation in ICU (P=0.001) in patients who had myasthenia gravis was more than patients without myasthenia gravis. These values were less in patients with myasthenia gravis and diaphragm plication than patients with myasthenia gravis and no diaphragm plication.

**Conclusion:**

Excision of the phrenic nerve in patients with myasthenia gravis associated with thymoma and phrenic nerve involvement is appropriate.

## INTRODUCTION

Thymoma is a thymic epithelial cell neoplasm that easily relapses and gives metastasis. Thymoma is the most common tumor of the anterior mediastinum (1,2). The most effective treatment of thymoma is complete removal of the thymus surgically (2,3). In 33% of those with advanced stages [III, IV] of thymoma, phrenic nerve involvement can be seen (3). Suggested treatment for patients with phrenic nerve involvement is unilateral excision of involved phrenic nerve. This procedure does not cause a significant problem in adults, and hemi-diaphragm paralysis causes 20 to 30 percent decrease in vital capacity and total lung capacity (2). In these patients, diaphragm plication is performed in addition to phrenic nerve excision. Plication of the diaphragm increases lung expansion and prevents paradoxical movement of the diaphragm (4–6). This procedure is very useful for patients who suffer from phrenic nerve injury and resultant shortness of breath (4,5).

There are concerns about the possibility of phrenic nerve injury in patients with bilateral phrenic nerve involvement, minimal involvement of phrenic nerve, people with respiratory problems before surgery, thymoma accompanied by myasthenia gravis and young people who need multiple chest surgery (3). Therefore, in these patients radiotherapy is used after thymectomy to preserve the phrenic nerve (3,7). Survival is the same in patients with excision of phrenic nerve in comparison with the patients without excision of phrenic nerve. Of course, in this method, there is the possibility of recurrence of thymoma (2, 3). There are measures to choose the appropriate method of treatment in patients with thymoma and phrenic nerve involvement. One of them is the presence or absence of myasthenia gravis in association with thymoma (2).

The aim of this study is to evaluate the effects of plication of the diaphragm on complication of phrenic nerve excision in thymoma patients with and without myasthenia gravis involving the phrenic nerve.

## MATERIALS AND METHODS

A retrospective cohort study was performed on 26 patients with thymoma; half of the patients had myasthenia gravis and the other half did not have myasthenia gravis. All patients underwent phrenic nerve resection due tumoral involvement. We performed diaphragm plication in 7 patients in each group with excision of phrenic nerve. These patients were evaluated based on variables including age, sex, clinical manifestations, blood loss during surgery, need for transfusion during surgery, ICU stay, intubation in ICU, short term complications, need for tracheostomy and mortality. Follow up of patients was performed for 14.6±3.5 month in all patients.

### Statistical analysis

Means and standard deviation are used for continuous variables. Frequencies and percentages are used for categorical variables.

ANOVA with post hoc was used to compare continuous variables between the four study groups. The Student's t test or the Mann-Whitney test was used for comparison of continuous variables between two reclassification groups. Fisher's exact test was used for categorical variables.

Statistical significancy was considered as p value of <0.05. The statistical analysis was performed using SPSS^®^ version 16 [IBM SPSS, Chicago, IL].

## RESULTS

**Figure 1.**
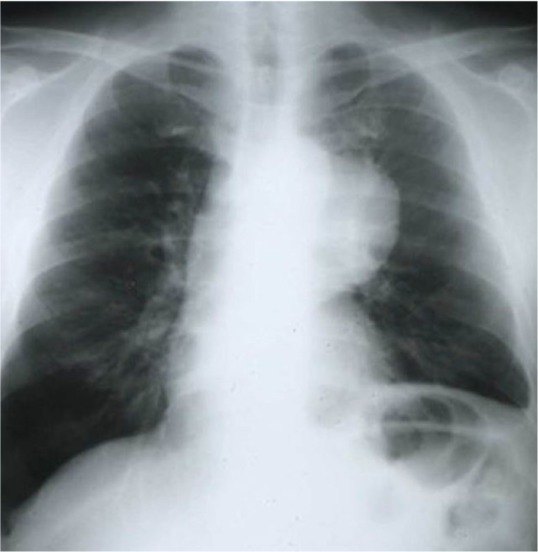
CXR of patient with thymoma and diaphragm elevation.

**Figure 2.**
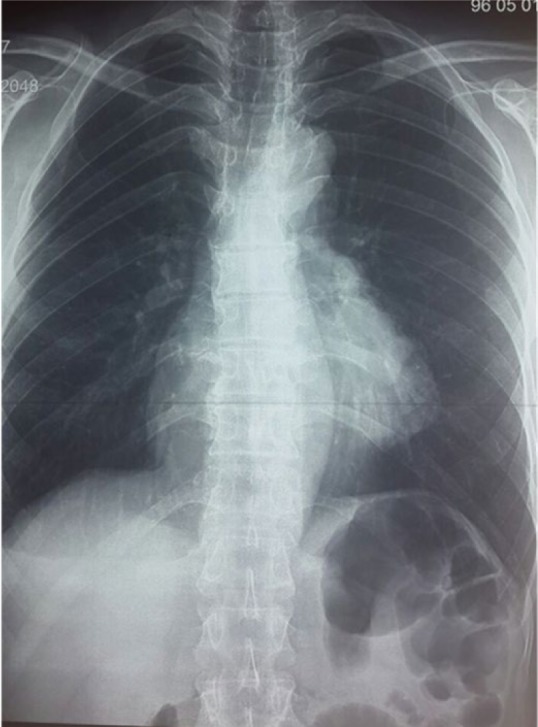
CXR of patients with thymoma with myasthenia gravis.

**Figure 3.**
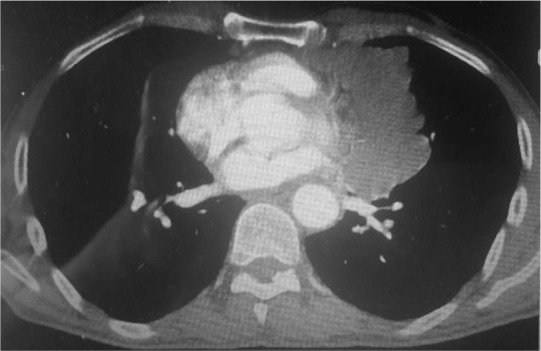
CT scan of patients with thymoma with myasthenia gravis.

**Figure 4.**
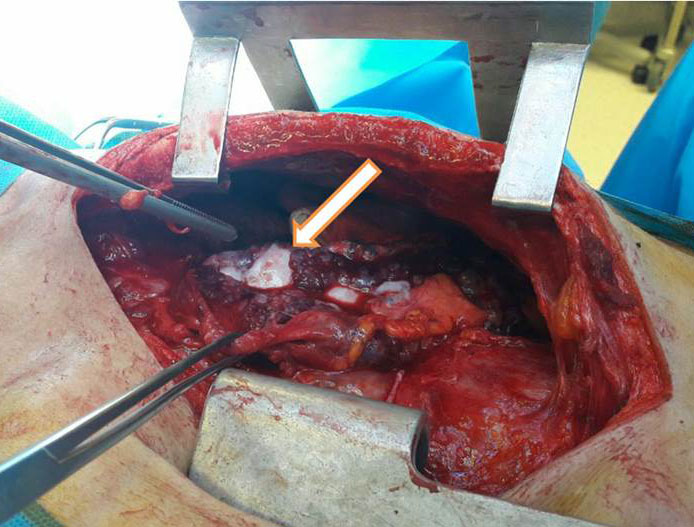
Intraoperative tumor view (white arrow show tumor with lung and pherenic nerve involvement).

**Figure 5.**
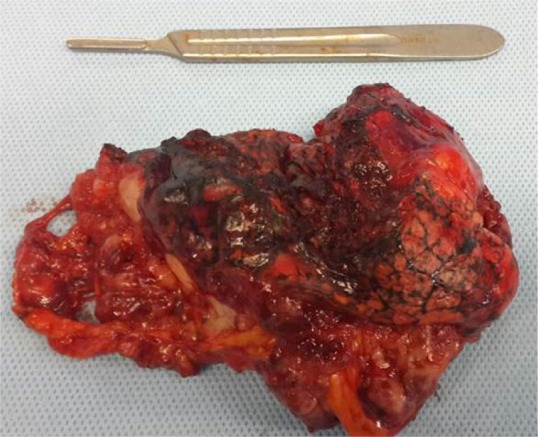
Tumor view after resection.

**Figure 6.**
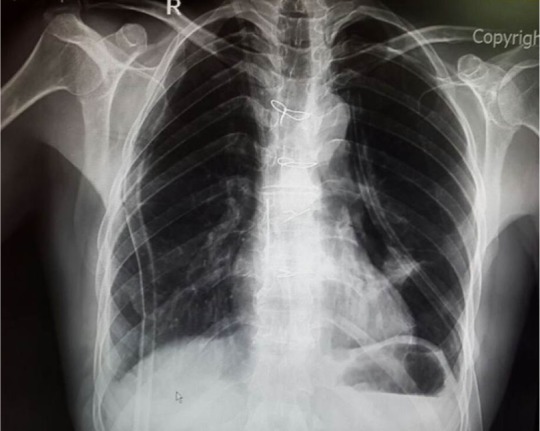
CXR of patients after surgery. Resection of tumor with phrenic nerve plus plication of diaphragm).

In this study, 26 patients with thymoma and phrenic nerve involvement were examined; 13 patients of whom did not have myasthenia gravis. 7 patients underwent thymoma resection with excision of phrenic nerve and plication of hemi-diaphragm [Group A1], but in the other 6 patients, we did not perform plication of diaphragm [Group A2]. Figure 1 shows CXR of patients with thymoma and phrenic nerve paralysis and diaphragm elevation. Figures 2 and 3 show CXR and CT-scan of another patient with thymoma with myasthenia gravis and phrenic nerve involvement. Figure 4 shows intraoperative view of tumor involvement of lung parenchyma and phrenic nerve. Figure 5 shows the resected tumor and lung parenchyma and phrenic nerve. Figure 6 shows CXR of patient after tumor and phrenic nerve resection with diaphragm plication.

13 patients had thymoma and myasthenia gravis. Seven of them underwent plication of diaphragm [Group B1] and for the other six patients, diaphragm plication was not performed [Group B2]. There was no significant difference in terms of age (P=0.943), blood loss during surgery (P=0.919), need for transfusion during surgery (P=0.791), short term complications (P=0.186), need for tracheostomy (P=0.209) and mortality (P=0.065) between these four groups (Table 1). There was no significant difference in terms of age (P=0.757), intraoperative blood loss (P=0.828), need for transfusion during surgery (P=0.999), short term complications (P=0.365), need for tracheostomy (P=0.580), mortality (P=0.203), duration of ICU stay (P=0.279) and duration of intubation in ICU (P=0.274) between the patients who underwent diaphragm plication [Groups A1, B1] and those without plication [Group A2, B2] (Table 2).

**Table 1.** Preoperative and postoperative data in all four groups

**Variables**		**Group A_1_**	**Group A_2_**	**Group B_1_**	**Group B_2_**	**P Value**
**Age(Year)**		52.71±4.6	51.66±5.3	50.00±2.3	53.83±5.9	0.943
**Blood loss during surgery(ml)**		290.00±43.6	310.00±52.3	318.57±48.1	280.00±20.1	0.919
**Intubation in ICU(day)**		2.14±0.4	2.83±0.3	9.28±0.5	12.83±0.8	0.001
**ICU stay(day)**		3.14±0.4	3.83±0.3	10.42±0.4	14.00±0.9	0.001
**Gender**	**Male**	5/6(83.3%)	5/7(71.4%)	5/6(83.3%)	6/7(85.7%)	0.907
	**Female**	1/6(16.7%)	2/7(28.6%)	1/6(16.7%)	1/7(14.3%)	
**Need for transfusion during surgery**		1/7(14.3%)	1/6(16.7%)	1/7(14.3%)	0/6(0.0%)	0.999
**Short term complications**		0/7(0.0%)	1/6(16.7%)	2/7(28.6%)	3/6(50.0%)	0.186
**Need for thoracostomy**		0/7(0.0%)	0/6(0.0%)	1/7(14.3%)	2/6(33.3%)	0.27
**Mortality**		0/7(0.0%)	0/6(0.0%)	0/7(0.0%)	2/6(33.3%)	0.09

**Table 2.** Preoperative and postoperative data in patients with and without plication

**Variables**		**Group A_1_,B_1_**	**Group A_2_,B_2_**	**P Value**
**Age(Year)**		51.35±9.3	52.75±13.2	0.757
**Blood loss during surgery(ml)**		304.28±117.7	295.00±93.9	0.828
**Intubation in ICU(day)**		5.71±3.8	7.83±5.4	0.274
**ICU stay(day)**		6.78±3.9	8.91±5.5	0.279
**Gender**	**Male**	11/14(78.6%)	10/12(83.3%)	0.999
	**Female**	3/14(21.4%)	2/12(16.7%)	
**Need for transfusion during surgery**		2/14(14.3%)	1/12(8.3%)	0.999
**Short term complications**		2/14(14.3%)	4/12(33.3%)	0.365
**Need for thoracostomy**		1/14(7.1%)	2/12(16.7%)	0.580
**Mortality**		0/14(0.0%)	2/12(16.7%)	0.203

The mean duration of ICU stay (P=0.001) and duration of intubation in ICU (P=0.001) was longer in patients with myasthenia gravis [Group B1, B2] in comparison to patients without myasthenia gravis [Group A1, A2], but there was no significant difference in terms of age (P=0.918), blood loss during surgery (P=0.971), need for transfusion during surgery (P=0.999), short term complications (P=0.160), need for tracheostomy (P=0.220), and mortality (P=0.480) between the patients with myasthenia gravis and patients without myasthenia gravis (Table 3). The mean duration of intubation [day] was the most in the group B2 and the least in group A1, but there was no significant difference between group A1 and A2 (P=0.816). Also there was no significant difference between group A1 and A2 in terms of ICU stay (P=813). Duration of ICU stay was the most in group B2 and the least for group A1. Although complication with significancy shows better result in non myasthenia group than with myasthenia group, better result shows in group with plication versus without plication in 2 groups without significancy.

**Table 3.** Preoperative and postoperative data in patients with and without Myasthenia gravis

**Variables**		**Group A_1_,A_2_**	**Group B_1_,B_2_**	**P Value**
**Age(Year)**		52.23±12.0	51.76±10.5	0.918
**Blood loss during surgery(ml)**		299.23±116.7	300.76±97.5	0.971
**Intubation in ICU(day)**		2.46±0.9	10.92±2.4	0.001
**ICU stay(day)**		3.46±0.9	12.07±2.4	0.001
**Gender**	**Male**	10/13(76.9%)	11/13(84.6%)	0.999
	**Female**	3/13(23.1%)	2/13(15.4%)	
**Need for transfusion during surgery**		2/13(15.4%)	1/13(7.7%)	0.999
**Short term complications**		1/13(7.7%)	5/13(38.5%)	0.160
**Need for thoracostomy**		0/13(0.0%)	3/13(23.1%)	0.220
**Mortality**		0/13(0.0%)	2/13(15.4%)	0.480

## DISCUSSION

The phrenic nerve is formed from C3,C4,C5 roots (8,9, 10). The phrenic nerve lies in front of anterior scalene muscle and before entering the thorax, it passes in front of the subclavian artery (11). It comes down in front of the pulmonary hilum between pericardium and parietal pleura. This nerve consists of sensory-motor and sympathetic fibers. This is the only motor nerve of the diaphragm and provides sensory fibers of the pericardium, mediastinal pleura and peritoneum of the diaphragm. This is the reason of importance of this nerve in respiratory cycles (9,10). So, this nerve should be preserved in thoracic surgery (12). Injury to this nerve results in hemi-diaphragm paralysis and paradoxical movement of the diaphragm and causes respiratory problems (8,9,11,13). Diaphragm paralysis following injury to this nerve is more harmful in pediatric population than in adults, because respiration is not exclusively dependent to diaphragm function in adults (13).

Schwartz and their colleagues evaluated 6 infants with diaphragmatic paralysis and severe breathing problems and following results were obtained using diaphragm plication. Respiratory function improvement was observed in 5 patients and in 2 patients diaphragmatic function returned to preoperative level. The result of this study demonstrated that diaphragm plication is a safe approach to improve breathing in children with paralysis of the diaphragm (14).

Higgs did diaphragm plication on 18 patients with diaphragm paralysis [18 patients with dyspnea and elevation of diaphragm on chest X-ray and paradoxical movement of diaphragm on ultrasound]. He concluded that values of FVC, FEV1, FRC1, and TRC were 10.1, 11.8, 16.9, and 9.2% at the beginning of the follow up and these values promoted to 11.8, 15.3, 26, and 13.3% at the end of the follow up (15).

In Freeman's study performed on 41 patients with diaphragm paralysis, diaphragm plication caused an increase of 19, 21, 23, and 19% in the values of mean forced vital capacity, FEV1, FRC, and TRC, respectively. They concluded that diaphragm plication for the unilateral diaphragm paralysis can promote pulmonary function test values and improve the functional condition in majority of the patients in long term. This is not true for the cases of morbid obesity or longstanding paralysis of diaphragm (16). The results of Welvaart's study showed increase in tidal volume and decrease in respiratory frequency after plication, but no change in maximal exercise capacity (6).

Jedrzejowicz and colleagues found that maximal static expiratory and inspiratory mouth pressure reduced in 16 of 17 patients with myasthenia gravis and also vital capacity was abnormal in 12 of the patients and transdiaphragmatic pressure reduced in 8 patients. There was no relationship between the grade of myasthenia gravis with the severity of dyspnea and muscle strength (17).

## CONCLUSION

Excision of the phrenic nerve in patients with myasthenia gravis associated with thymoma and phrenic nerve involvement is appropriate.
